# Maternal Thyroid Hormone Evaluation in Women With Gestational Diabetes Mellitus in the Second Trimester

**DOI:** 10.7759/cureus.98107

**Published:** 2025-11-29

**Authors:** Divya Christy, Jahnavi Chandrasekar

**Affiliations:** 1 Obstetrics and Gynaecology, Sree Balaji Medical College and Hospital, Chennai, IND

**Keywords:** anti-thyroid peroxidase antibodies, gdm- gestational diabetes mellitus, hypothyroxinemia, pregnancy outcomes, thyroid dysfunction

## Abstract

Background

Thyroid dysfunction during pregnancy has emerged as an important yet under-recognized contributor to adverse maternal and fetal outcomes. Both gestational diabetes mellitus (GDM) and thyroid disease share overlapping metabolic and hormonal pathways that may synergistically amplify obstetric risk. In developing countries such as India, where the dual burden of iodine deficiency and gestational dysglycemia persists, understanding this association is essential for optimizing antenatal care. This study aimed to evaluate the prevalence, determinants, and perinatal consequences of thyroid dysfunction among women with GDM in the second trimester.

Methods

A hospital-based cross-sectional analytical study was conducted among 105 pregnant women aged 18-40 years diagnosed with GDM using the DIPSI single-step test (2-hour plasma glucose ≥140 mg/dL after 75 g glucose). Women with pre-existing thyroid or systemic illness were excluded. Serum thyroid-stimulating hormone (TSH), free thyroxine (FT4), and anti-thyroid peroxidase (anti-TPO) antibodies were measured using the electrochemiluminescence immunoassay method. Thyroid dysfunction was classified using ATA pregnancy-specific reference ranges. Maternal demographic, biochemical, and obstetric parameters were analyzed using SPSS version 26. Logistic regression identified predictors of thyroid dysfunction, with *p* < 0.05 considered significant.

Results

Thyroid dysfunction was detected in 11.4% of GDM women, comprising mainly subclinical hypothyroidism. Women with thyroid dysfunction were significantly older (30.2 ± 4.8 years) and had higher BMI (28.5 ± 4.2 kg/m²) compared to euthyroid women (*p* = 0.042 and *p* = 0.008, respectively). Family history of thyroid disorder (41.7% vs 12.9%; p = 0.012) and anti-TPO positivity (33.3% vs 5.4%; p = 0.003) were strongly associated. On multivariate analysis, anti-TPO positivity (aOR 6.78; p = 0.016) and familial thyroid history (aOR 4.12; p = 0.047) independently predicted dysfunction. Insulin therapy was required more often (50% vs 24.7%), indicating greater metabolic derangement. Adverse neonatal outcomes were higher - macrosomia (41.7% vs 19.4%; p = 0.048) and NICU admission (33.3% vs 12.9%; p = 0.045) - among women with thyroid dysfunction.

Conclusion

The coexistence of thyroid dysfunction and GDM in mid-pregnancy is clinically significant and frequently autoimmune in nature. Routine screening for thyroid function and anti-TPO antibodies in GDM women can facilitate early diagnosis, prevent perinatal complications, and improve maternal-fetal outcomes through timely intervention.

## Introduction

Pregnancy is a period marked by extensive physiological adaptation, involving complex metabolic, hormonal, and immunological adjustments that support fetal growth and safeguard maternal health. Among the endocrine systems undergoing significant modulation during gestation, the thyroid gland plays a central role. Thyroid hormones, primarily thyroxine (T4) and triiodothyronine (T3), regulate basal metabolic rate, thermogenesis, carbohydrate utilization, lipid turnover, and protein synthesis, all of which contribute to the altered metabolic demands of pregnancy. The pooled prevalence of hypothyroidism among pregnant women was estimated to be 11.07% [1]. Even subtle disturbances in maternal thyroid function can therefore influence insulin sensitivity, glucose regulation, and fetal neurodevelopment. This interrelationship has led to growing interest in the association between maternal thyroid dysfunction and gestational diabetes mellitus (GDM), especially in countries such as India, where both thyroid disorders and metabolic diseases are increasingly prevalent [2,3].

GDM, defined as carbohydrate intolerance of variable severity with onset or first recognition during pregnancy, is a major contributor to maternal and neonatal morbidity. It affects approximately one in seven pregnancies globally, with disproportionately higher burden in South and Southeast Asia, where urbanization, sedentary behavior, and dietary transitions have accelerated metabolic risk profiles [4]. In India, the prevalence of gestational diabetes mellitus (GDM) varies widely, from about 10% to 35%, a difference partly attributed to socioeconomic disparities, dietary and lifestyle patterns, and regional differences in iodine sufficiency, since iodine deficiency can impair thyroid function, which in turn affects glucose metabolism and increases the risk of GDM [5]. This dual burden of thyroid dysfunction and GDM has raised clinical concerns, particularly since both conditions can have synergistic adverse effects on pregnancy outcomes.

The biological basis linking thyroid regulation and glucose metabolism is multifaceted. Thyroid hormones influence pancreatic beta-cell function, hepatic glucose production, and peripheral glucose uptake. Consequently, reductions in circulating thyroid hormone levels may impair insulin responsiveness and contribute to glucose intolerance [6]. Evidence from a case-control study in North India demonstrated that pregnant women with elevated thyroid-stimulating hormone (TSH) and reduced free thyroxine (FT4) in the second trimester were more likely to develop GDM, suggesting a pathogenic association between hypothyroid states and gestational dysglycemia [4]. Similar observations were reported in a South American cohort where first- and second-trimester thyroid profiles showed predictive value for GDM risk when analyzed using machine-learning models, reinforcing the utility of endocrine biomarkers in early pregnancy screening [5].

Further longitudinal research in East Asian populations demonstrated that maternal thyroid derangements were independently associated not only with increased GDM risk but also with a spectrum of adverse perinatal outcomes, including macrosomia, preeclampsia, and higher rates of cesarean delivery [3]. This relationship appears to be trimester-specific. For example, a Turkish cohort revealed that women in the upper tertile of TSH levels during the second trimester exhibited a significantly higher probability of developing GDM, even when FT4 concentrations remained within standard reference limits [7]. Such findings imply that conventional trimester-adjusted thyroid reference intervals may not adequately reflect clinically meaningful variations in thyroid function during pregnancy.

The interplay of thyroid hormones with maternal adiposity further contributes to the complexity of metabolic regulation. Mediation analyses have shown that thyroid hormones may modify the association between pre-pregnancy body-mass index and GDM risk, indicating that suboptimal thyroid function may exacerbate metabolic stress in overweight or obese women [8]. Nutritional factors, particularly iodine intake, also modulate thyroid-metabolic interactions. A European cohort demonstrated that low iodine intake was associated with altered thyroid function among women with GDM, suggesting that micronutrient insufficiency contributes to endocrine-metabolic dysregulation during pregnancy [9]. Moreover, earlier European registry data indicated that even euthyroid women with TSH levels between 2.5 and 5.0 mIU/L experienced higher rates of pregnancy loss and GDM compared to those with lower TSH levels, raising questions regarding the applicability of traditional upper-limit thresholds in pregnancy [10].

There is also evidence that immunological processes play a role in the overlap between thyroid dysfunction and GDM. For example, a Middle Eastern study has shown increased prevalence of hypothyroxinemia and autoimmune thyroid disease, particularly anti-thyroid peroxidase antibody positivity, in women with GDM [11]. European and Nordic longitudinal studies have similarly demonstrated that declining FT4 levels across pregnancy parallel progressive insulin resistance, reinforcing physiologic interactions between thyroid and glycemic control [12]. Large multicenter investigations in the United States further found that FT4 in early pregnancy was an independent predictor of GDM development [13].

Recent findings from China expanded this relationship by demonstrating that elevated free triiodothyronine (FT3) levels were closely associated with gestational dysglycemia, suggesting FT3 as a meaningful early biomarker of metabolic dysfunction in pregnancy [14]. The present hospital-based observational study undertaken at Sree Balaji Medical College and Hospital, Chennai, seeks to evaluate maternal thyroid hormone status among women with gestational diabetes in the second trimester. By focusing on this critical period (when both thyroid and placental hormonal shifts peak), the study aims to determine the prevalence and pattern of overt and subclinical thyroid dysfunction among women already diagnosed with GDM and to correlate these findings with maternal and neonatal outcomes.

## Materials and methods

Study design and setting

This hospital-based observational analytical study was conducted in a tertiary-level obstetric center catering to both urban and semi-urban populations in and around Chennai. The institution provides comprehensive antenatal, intrapartum, and neonatal services and receives referrals from surrounding primary and secondary care facilities, thereby supporting a diverse patient base. The present investigation focused on assessing thyroid hormone status among women diagnosed with gestational diabetes mellitus (GDM) during the second trimester (13-28 weeks) and on determining the prevalence and perinatal implications of coexisting thyroid dysfunction in this population. Ethical clearance for the study was obtained from the Institutional Human Ethics Committee, Sree Balaji Medical College and Hospital (Approval No: 002/SBMC/IHEC/2023/2502). Written informed consent was secured from all eligible participants prior to enrollment, ensuring adherence to ethical guidelines.

Study duration and design

The study followed a cross-sectional analytical design and was conducted over an 18-month period, extending from October 2023 to March 2025. This design enabled simultaneous assessment of thyroid profiles and glycemic parameters during the same phase of pregnancy, thereby facilitating direct correlation between maternal thyroid function and metabolic status at the time of diagnosis and management of GDM.

Study population and eligibility criteria

The study population included parturients aged 18-40 years with singleton pregnancy who attended antenatal clinics or were admitted for obstetric care at the study site. Eligibility was determined on the basis of GDM diagnosis using the Diabetes in Pregnancy Study Group of India (DIPSI) single-step test. The DIPSI protocol, widely used in Indian antenatal practice, involves the administration of 75 g of oral glucose, irrespective of the last meal, followed by measurement of venous plasma glucose after two hours [15]. A value ≥140 mg/dL was considered confirmatory of GDM.

To minimize confounding, pregnant women with pre-existing thyroid disease (on treatment or previously diagnosed), overt diabetes mellitus, multiple gestations, and chronic systemic illnesses affecting hepatic, renal, cardiovascular, or endocrine function were excluded. These exclusion criteria ensured that observed thyroid and metabolic variations could be attributed primarily to gestational changes rather than underlying chronic disease states.

Sample size determination and sampling technique

The sample size was calculated using Dobson’s single-proportion formula,

\[n = \frac{Z^2 \cdot p(1 - p)}{d^2}\]

where Z represents the standard normal deviate at 95% confidence (1.96), p the estimated prevalence of thyroid dysfunction among women with GDM (11.05% based on Sahu et al. [16]), and d the acceptable relative precision (0.06). The computed sample size was 95. To compensate for potential non-response or incomplete data, an additional 10% was added, yielding a final required sample size of 105 participants. A consecutive sampling method was used to ensure uniform recruitment and minimize selection bias.

Data collection and clinical assessment

After consent, participants’ demographic and obstetric details were recorded using a structured, pre-validated proforma. Variables collected included maternal age, gravidity, parity, gestational age, pre-pregnancy body weight, height, and body mass index (BMI), along with weight gain during pregnancy. Family history of thyroid disease or diabetes was specifically documented due to its potential predictive relevance.

Clinical evaluation included general physical examination, systemic assessment, and obstetric evaluation. Particular attention was given to the thyroid gland for evidence of enlargement, nodules, or tenderness. Blood pressure and anthropometric measurements were taken using standardized and calibrated instruments to minimize measurement error.

Laboratory analysis

Venous blood samples were obtained under aseptic precautions following an overnight fast. Fasting plasma glucose (FPG) and two-hour postprandial glucose (PPG) levels were measured using the enzymatic glucose oxidase-peroxidase method. Thyroid function assessment included quantification of thyroid-stimulating hormone (TSH) and free thyroxine (FT4) levels, while anti-thyroid peroxidase (anti-TPO) antibody testing was conducted to assess autoimmune involvement. All assays were performed using Electrochemiluminescence Immunoassay (ECLIA) in a quality-controlled laboratory environment with regular internal and external proficiency validation.

Classification of thyroid status

Thyroid status was classified according to pregnancy-specific reference ranges recommended by the American Thyroid Association (ATA), which account for the physiological alterations in thyroid hormone levels during gestation [17]. Women with thyroid-stimulating hormone (TSH) and free thyroxine (FT4) values within the trimester-adjusted reference intervals were considered euthyroid. Subclinical hypothyroidism was defined by elevated TSH with normal FT4, whereas overt hypothyroidism was diagnosed when both TSH was elevated, and FT4 was reduced. Isolated hypothyroxinemia refers to cases where FT4 was decreased despite a normal TSH level, reflecting impaired thyroid hormone availability without pituitary response. Additionally, autoimmune thyroiditis was identified based on positive anti-thyroid peroxidase (anti-TPO) antibodies, regardless of whether TSH and FT4 values were within normal pregnancy-specific limits. This classification allowed precise differentiation of thyroid functional states among the study population.

Maternal and neonatal outcome assessment

Maternal outcomes assessed included hypertensive disorders of pregnancy, pre-eclampsia, polyhydramnios, preterm labor, and mode of delivery. Neonatal outcomes included birth weight, Apgar score at five minutes, neonatal hypoglycemia, macrosomia, and requirement for NICU admission. All newborns were evaluated by a pediatrician immediately after birth.

Statistical analysis

Data were entered into Microsoft Excel and analyzed using SPSS version 26.0. Continuous variables were expressed as mean ± standard deviation, and categorical variables as frequencies and percentages. The Shapiro-Wilk test was used to assess normality. Intergroup comparisons utilized the independent sample t-test for continuous variables and the chi-square test or Fisher’s exact test for categorical variables. A p-value <0.05 was considered statistically significant.

To identify predictors of thyroid dysfunction among GDM women, univariate logistic regression was first applied to estimate crude odds ratios. Variables with p < 0.10 in univariate analysis were included in multivariate logistic regression, yielding adjusted odds ratios (aOR) with 95% confidence intervals, while the Hosmer-Lemeshow test evaluated model fit.

Quality assurance and ethical considerations

Stringent quality control procedures were applied throughout data collection and laboratory processing. Instruments were calibrated regularly, laboratory assays were replicated in 10% of samples, and double data entry minimized transcription errors. Confidentiality was maintained at all stages in accordance with ethical standards and the Declaration of Helsinki.

## Results

**Figure 1:**
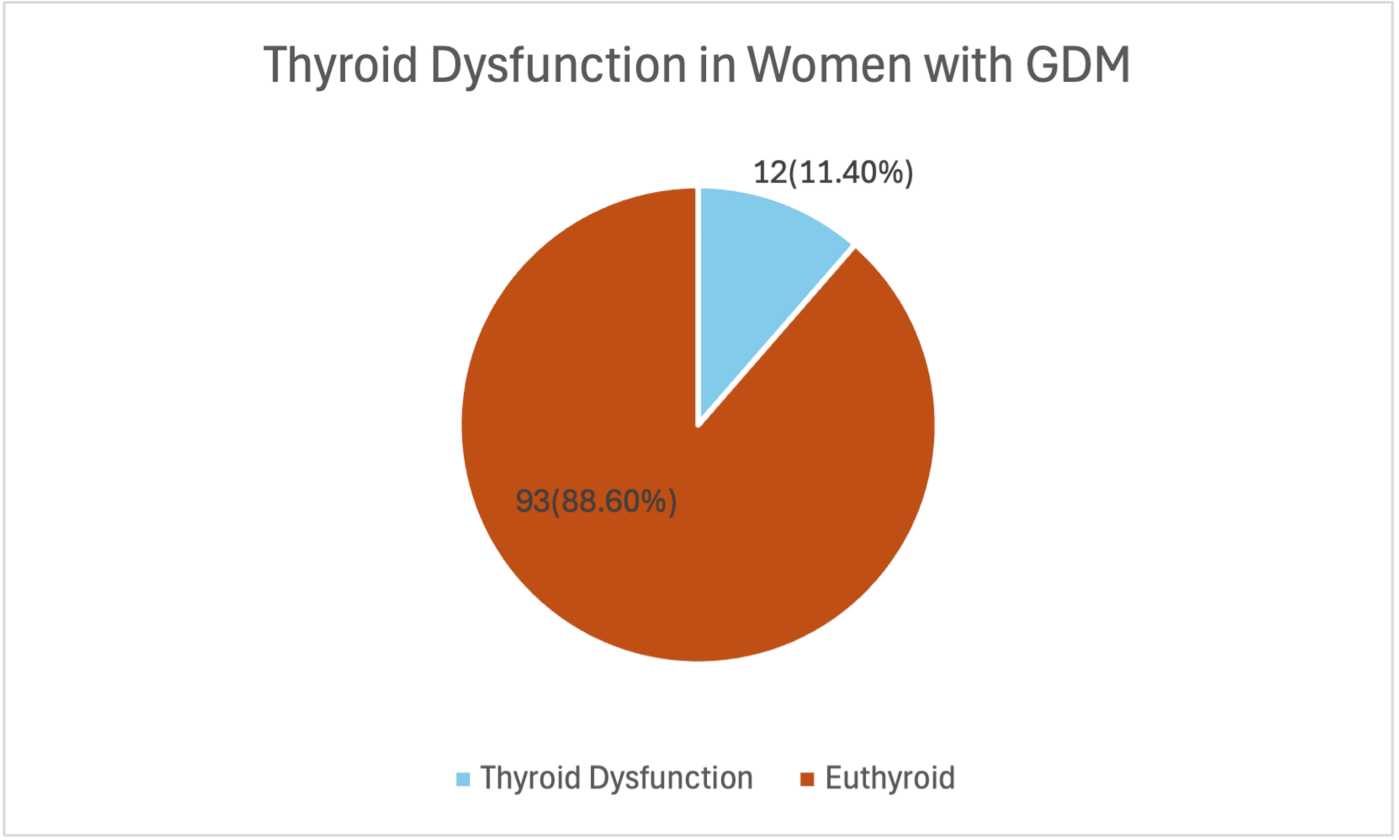
Distribution of thyroid dysfunction among the study participants in second trimester (n=105)

This hospital-based observational study investigated the prevalence, predictors, and obstetric implications of thyroid dysfunction among women with GDM during the second trimester at Sree Balaji Medical College and Hospital, Chennai. Figure 1 shows that 12 out of 105 women (11.4%) with GDM had thyroid dysfunction (of whom 3 (25%) had overt and 9 (75%) had subclinical hypothyroidism), while 93 (88.6%) were euthyroid. The study compared demographic, clinical, biochemical, and perinatal outcomes between women with thyroid dysfunction and those who were euthyroid, aiming to identify significant correlates.

Among women with thyroid dysfunction, 5 (41.7%) were primigravida and 7 (58.3%) were multigravida, whereas in the euthyroid group, 52 (55.9%) were primigravida and 41 (44.1%) were multigravida. A family history of thyroid disorder was present in 5 women (41.7%) in the thyroid dysfunction group compared to 12 women (12.9%) in the euthyroid group, indicating a significant association. Similarly, 8 women (66.7%) with thyroid dysfunction and 42 women (45.2%) in the euthyroid group reported a family history of diabetes. Regarding treatment patterns, 6 women (50.0%) in the thyroid dysfunction group required insulin therapy compared to 23 women (24.7%) in the euthyroid group, while lifestyle modification alone was sufficient for 6 women (50.0%) with thyroid dysfunction and 70 women (75.3%) who were euthyroid. This distribution suggests that GDM women with thyroid dysfunction more frequently require pharmacological intervention (Table 1).

**Table 1:** Association between demographic and clinical characteristics of study participants stratified by thyroid status (n=105) Chi-square/independent sample t-test *p-value <0.05 is significant

Characteristic	Thyroid Dysfunction (n=12)	Euthyroid (n=93)	Test Statistic (t / χ²)	p-value
Maternal age (years), mean ± SD	30.2 ± 4.8	28.1 ± 4.0	*t* ≈ 2.1	0.042*
Parity, n (%)				
Primigravida	5 (41.7%)	52 (55.9%)	χ² ≈ 1.0	0.312
Multigravida	7 (58.3%)	41 (44.1%)		
BMI at booking (kg/m²), mean ± SD	28.5 ± 4.2	25.8 ± 3.6	*t* ≈ 2.7	0.008*
Weight gain (kg), mean ± SD	4.1 ± 1.2	3.9 ± 1.0	*t* ≈ 0.75	0.451
Gestational age at enrollment (weeks), mean ± SD	26.3 ± 2.0	26.8 ± 2.1	*t* ≈ 0.90	0.378
Family history of diabetes, n (%)	8 (66.7%)	42 (45.2%)	χ² ≈ 2.1	0.149
Family history of thyroid disorder, n (%)	5 (41.7%)	12 (12.9%)	χ² ≈ 6.4	0.012*
Fasting blood glucose (mg/dL), mean ± SD	102.3 ± 12.4	98.7 ± 10.9	*t* ≈ 1.2	0.231
Postprandial blood glucose (mg/dL), mean ± SD	148.5 ± 15.2	142.1 ± 13.8	*t* ≈ 1.7	0.089
HbA1c (%), mean ± SD	5.9 ± 0.6	5.7 ± 0.5	*t* ≈ 1.4	0.156
GDM treatment, n (%)				
Lifestyle modification	6 (50.0%)	70 (75.3%)	χ² ≈ 3.1	0.078
Insulin	6 (50.0%)	23 (24.7%)		

Univariate logistic regression identified several potential determinants of thyroid dysfunction. Maternal age ≥ 30 years (OR 3.21; p = 0.045) and BMI ≥ 27 kg/m² (OR 4.13; p = 0.021) were significant risk factors. A positive family history of thyroid disorder showed the strongest unadjusted association (OR 4.92; p = 0.009), confirming hereditary predisposition. Anti-TPO antibody positivity showed markedly increased odds of thyroid dysfunction (OR 8.00; p = 0.003), highlighting the autoimmune nature of the disease in this cohort. Clinical complications such as pre-eclampsia and oligohydramnios were more frequent among women with thyroid dysfunction, although these trends did not achieve statistical significance, likely due to small subgroup size. Insulin therapy was required in 50% of women with thyroid dysfunction compared to 24.7% of euthyroid women (OR 3.04; p = 0.072), suggesting that concomitant thyroid impairment may exacerbate glycemic instability in GDM (Table 2).

**Table 2:** Univariate analysis of factors associated with thyroid dysfunction in GDM (n=105) *p-value<0.05 is significant GDM: gestational diabetes mellitus; BMI: body mass index; IUGR: intrauterine growth restriction; anti-TPO: anti-thyroid peroxidase

Factor	Thyroid Dysfunction (n=12) n (%)	Euthyroid (n=93) n (%)	Unadjusted OR (95% CI)	p-value
Age				
≥30 years	7 (58.3)	28 (30.1)	3.21 (0.95–10.85)	0.045*
<30 years	5 (41.7)	65 (69.9)	Ref	
BMI				
≥27 kg/m²	8 (66.7)	30 (32.3)	4.13 (1.12–15.24)	0.021*
<27 kg/m²	4 (33.3)	63 (67.7)	Ref	
Gravida				
Multigravida	7 (58.3)	41 (44.1)	1.78 (0.52–6.12)	0.356
Primigravida	5 (41.7)	52 (55.9)	Ref	
Family history of diabetes				
Yes	8 (66.7)	42 (45.2)	2.33 (0.65–8.35)	0.178
No	4 (33.3)	51 (54.8)	Ref	
Family history of thyroid disorder				
Yes	5 (41.7)	12 (12.9)	4.92 (1.28–18.92)	0.009*
No	7 (58.3)	81 (87.1)	Ref	
Pre-eclampsia				
Yes	3 (25.0)	8 (8.6)	3.44 (0.76–15.62)	0.092
No	9 (75)	85 (91.4)	Ref	
Oligohydramnios				
Yes	2 (16.7)	5 (5.4)	3.47 (0.59–20.45)	0.156
No	10 (83.3)	88 (94.6)	Ref	
IUGR				
Yes	1 (8.3)	4 (4.3)	2.00 (0.21–19.23)	0.512
No	11 (91.7)	89 (95.7	Ref	
Anti-TPO				
Positive	4 (33.3)	5 (5.4)	8.00 (1.79–35.74)	0.003*
Negative	8 (66.7)	88 (94.6)	Ref	
GDM treatment				
Insulin therapy required	6 (50.0)	23 (24.7)	3.04 (0.86–10.75)	0.072
Lifestyle only	6 (50)	70 (75.3)	Ref	

Table 3 shows that after adjusting for confounders, two independent predictors of thyroid dysfunction persisted. A family history of thyroid disorder (adjusted OR 4.12; p = 0.047) remained a significant determinant, and Anti-TPO antibody positivity independently predicted thyroid dysfunction (adjusted OR 6.78; p = 0.016). Although higher BMI (≥ 27 kg/m²) and age ≥ 30 years showed elevated odds, their associations lost statistical significance after adjustment, suggesting that familial predisposition and autoimmunity are stronger determinants than anthropometric factors alone.

**Table 3:** Multivariate logistic regression analysis for predictors of thyroid dysfunction in women with GDM (n=105) *p-value<0.05 is significant GDM: gestational diabetes mellitus; anti-TPO: anti-thyroid peroxidase

Predictor	Adjusted OR (95% CI)	p-value
Age		
≥30 years	2.85 (0.78–10.42)	0.112
<30 years	Ref	
BMI		
≥27 kg/m²	3.21 (0.82–12.58)	0.094
<27 kg/m²	Ref	
Family history of thyroid disorder		
Yes	4.12 (1.02–16.68)	0.047*
No	Ref	
Anti-TPO		
Positive	6.78 (1.45–31.72)	0.016*
Negative	Ref	

Among the maternal outcomes, cesarean section was more frequent in the thyroid dysfunction group, occurring in 8 out of 12 women (66.7%), compared to 42 out of 93 women (45.2%) in the euthyroid group, although this difference was not statistically significant. Similarly, hypertensive disorders and polyhydramnios were observed more often in women with thyroid dysfunction, at 4 (33.3%) and 3 (25.0%), respectively, compared to 15 (16.1%) and 12 (12.9%) among euthyroid women. Preterm labor was also slightly higher among women with thyroid dysfunction at 2 (16.7%) versus 8 (8.6%) in the euthyroid group. With respect to neonatal outcomes, macrosomia (birth weight >4 kg) was significantly more common in neonates born to mothers with thyroid dysfunction, at 5 (41.7%), compared to 18 (19.4%) in the euthyroid group (p = 0.048). Additionally, NICU admission was required in 4 (33.3%) infants of thyroid dysfunction mothers versus 12 (12.9%) in the euthyroid group (p = 0.045). Although the rates of neonatal hypoglycemia and low Apgar scores were higher in the thyroid dysfunction group, these differences were not statistically significant. Overall, these findings suggest that thyroid dysfunction in GDM may be associated with more adverse neonatal outcomes, particularly macrosomia and increased NICU admissions (Table 4).

**Table 4:** Comparison of maternal and neonatal outcomes by thyroid status Chi-square/ independent sample t-test *p-value<0.05 is significant

Outcome	Thyroid Dysfunction n (%) (n=12)	Euthyroid n (%) (n=93)	Test Statistic (χ² / Fisher’s Exact)	p-value
Maternal outcomes				
Cesarean section	8 (66.7%)	42 (45.2%)	χ² ≈ 2.0	0.156
Hypertensive disorders	4 (33.3%)	15 (16.1%)	χ² ≈ 2.5	0.118
Polyhydramnios	3 (25.0%)	12 (12.9%)	χ² ≈ 1.7	0.201
Preterm labor	2 (16.7%)	8 (8.6%)	χ² ≈ 1.0	0.312
Neonatal outcomes				
Birth weight >4 kg (macrosomia)	5 (41.7%)	18 (19.4%)	χ² ≈ 4.0	0.048*
Apgar Score <7 at 5 min	2 (16.7%)	4 (4.3%)	χ² ≈ 2.5	0.112
NICU admission	4 (33.3%)	12 (12.9%)	χ² ≈ 4.1	0.045*
Neonatal hypoglycemia	3 (25.0%)	10 (10.8%)	χ² ≈ 2.0	0.156

## Discussion

In this study, thyroid dysfunction was identified in a subset of women with gestational diabetes mellitus (GDM), with subclinical hypothyroidism being the most frequent abnormality, followed by overt hypothyroidism. The presence of thyroid dysfunction in GDM highlights the intricate endocrine interplay between thyroid hormone regulation and maternal glucose metabolism. Women exhibiting thyroid dysfunction tended to be older (>30 years) and had a higher body mass index at booking compared to euthyroid women; women having thyroid dysfunction had higher adiposity and metabolic abnormalities. A strong association with family history of thyroid disease and a higher prevalence of anti-thyroid peroxidase (anti-TPO) antibody positivity further supports the role of underlying genetic and autoimmune mechanisms in the manifestation of thyroid dysfunction among women with GDM.

Multivariate analysis in this study confirmed that autoimmune thyroiditis and family history of thyroid disease were independent predictors of thyroid dysfunction. This finding is consistent with the broader understanding that pregnancy-induced immunological modulation can unmask latent thyroid autoimmunity. Though it was not statistically significant, a higher proportion of women with thyroid dysfunction required insulin therapy rather than lifestyle modification alone, suggesting that thyroid abnormalities may exacerbate insulin resistance and increase metabolic load during pregnancy

Maternal outcomes displayed trends toward less favorable profiles among women with thyroid dysfunction, particularly with respect to hypertensive disorders. Although statistical significance was not observed, the direction of association aligns with evidence that thyroid hormone abnormalities may influence vascular reactivity and endothelial function, thereby contributing to the development of hypertensive complications [14,16]. Neonatal outcomes were more distinctly impacted, with a higher likelihood of macrosomia and the need for neonatal intensive care unit (NICU) support among infants born to mothers with thyroid dysfunction. These findings reinforce the concept that thyroid hormone insufficiency can exacerbate fetal growth alterations, potentially mediated through placental glucose transport mechanisms.

The findings of this study align with several international reports. Iijima, in a study from Japan, highlighted the diagnostic challenges associated with thyroid function assessment in early pregnancy, where gestational transient thyrotoxicosis may mask emerging hypothyroid states [18]. Unlike that study, which focused on early gestational physiology, the present work assessed thyroid function in the second trimester, a period characterised by stabilized thyroid-stimulating hormone (TSH) homeostasis, thereby reducing the likelihood of misclassification. This distinction supports the reliability of the observed prevalence of thyroid dysfunction in the present cohort.

Wang et al. in China demonstrated that alterations in thyroid hormone indices in early pregnancy, combined with dyslipidemia, contributed to increased GDM risk [19]. The current findings support the mechanistic plausibility of this association, as women with thyroid dysfunction exhibited higher body mass indices and a greater requirement for insulin therapy. Although lipid profiles were not measured in the present study, the observed metabolic burden is consistent with the hypothesis that thyroid hormone insufficiency may intensify insulin resistance through adipose-derived metabolic pathways.

Jia et al. reported that isolated maternal hypothyroxinemia was linked to increased risk of macrosomia and obstetric intervention [20]. The present study similarly observed greater fetal overgrowth among pregnancies with thyroid dysfunction. These parallels underscore that even mild reductions in circulating thyroid hormones may have clinically significant effects on fetal growth dynamics. Meanwhile, Ramezani Tehrani and colleagues demonstrated that isolated hypothyroxinemia increased NICU admissions and adverse perinatal outcomes [21], which correlates closely with the higher NICU admission rates observed here. 

Further support comes from large-scale cohort studies such as that by Huang et al., who reported that high TSH and low free thyroxine in pregnancy increased the risk of GDM regardless of baseline maternal body size [22]. The present findings mirror this endocrine-metabolic pattern, reinforcing the consistency of this association across Asian populations. Additionally, Zhang et al. demonstrated that thyroid function influences gestational lipid metabolism, thereby shaping insulin resistance [23]; although lipid measures were not included in this study, the direction of effect remains compatible with their proposed mechanistic model.

Meta-analytic evidence by Wang et al. showed that even high-normal TSH and low-normal free thyroxine values were associated with preeclampsia and GDM [24], reflecting a continuum of metabolic risk rather than a binary classification based on reference ranges. The observed trend toward hypertensive disorders in thyroid dysfunction pregnancies in this study aligns with this interpretation. Sun et al. further demonstrated that pre-pregnancy obesity modifies the association between thyroid function and GDM risk [25], and the present findings similarly suggest that adiposity amplifies the metabolic effects of thyroid hormone imbalance.

The large individual participant dataset analyzed by Osinga et al. confirmed the independent association between thyroid dysfunction and GDM across diverse populations [26]. The present study not only supports these biochemical associations but also extends the understanding by linking thyroid dysfunction to clinically meaningful neonatal outcomes. Shoshan and colleagues demonstrated alterations in neonatal thyroid adaptation among infants born to GDM mothers [27], aligning with the increased neonatal morbidity observed in this study. Pinto et al. emphasized the autoimmune dimension of thyroid-GDM interaction [28], which is strongly supported by the independent association of anti-TPO positivity observed here. Findings from Safian et al. further reinforce the link between thyroid dysfunction, increased insulin requirement, and long-term risk of thyroid disease postpartum [29], indicating clinical significance beyond pregnancy. Finally, Chen et al. showed that hypothyroid GDM women exhibit greater metabolic dysregulation [30], consistent with the greater insulin requirement observed in this study.

The study’s limitations include its modest sample size and cross-sectional design, which preclude causal inference. Thyroid assessment was restricted to the second trimester, and postpartum follow-up was not conducted. Lipid profiles, iodine status, and insulin resistance indices were not measured, limiting metabolic mechanistic insight. Additionally, due to the study's single-center and hospital-based design, generalizability may be constrained.

The findings highlight the importance of incorporating routine thyroid function screening, including anti-TPO testing, into the evaluation of women with GDM. Early identification and management of thyroid dysfunction may improve glycemic control, reduce dependence on insulin therapy, and mitigate risks of macrosomia and neonatal morbidity. Integrating thyroid assessment into antenatal protocols, particularly in regions with high metabolic and autoimmune disease burden, may therefore enhance maternal and neonatal health outcomes.

## Conclusions

The present study demonstrated that thyroid dysfunction, predominantly subclinical hypothyroidism, was present in 11.4% of women with GDM and was significantly associated with advancing maternal age, higher body mass index, family history of thyroid disorder, and anti-TPO antibody positivity. Women with thyroid dysfunction exhibited greater insulin requirement and a higher incidence of adverse perinatal outcomes, including macrosomia and NICU admissions, compared with euthyroid GDM women. Anti-TPO positivity and family history of thyroid disease emerged as independent predictors, underscoring the autoimmune contribution to thyroid impairment in GDM. These findings reinforce the growing global evidence that thyroid and glucose metabolism are closely interlinked during pregnancy, and even subtle thyroid abnormalities may exacerbate insulin resistance and metabolic stress. Routine thyroid function testing, including antibody screening, during the second trimester in GDM pregnancies can facilitate early identification and management of high-risk women. Incorporating such evaluation into standard antenatal protocols could improve metabolic stability, optimize glycemic control, and enhance neonatal outcomes. The study highlights the need for longitudinal multicentric research to explore causal pathways and postpartum thyroid evolution, paving the way for integrated maternal endocrine care strategies in high-risk populations.
